# Targeted Modulation of Mitochondrial Oxidative Stress Ameliorates 5-Fluorouracil-Induced Renal Injury in BALB/c Mice

**DOI:** 10.1155/omcl/8892026

**Published:** 2025-03-06

**Authors:** Prasad Kisan Tambe, Maya P. Shetty, Komal Rana, Sanjay Bharati

**Affiliations:** ^1^Department of Nuclear Medicine, Manipal College of Health Professions, Manipal Academy of Higher Education, Manipal, Karnataka, India; ^2^Manipal Government of Karnataka Bioincubator Advanced Research Centre, Manipal Academy of Higher Education, Manipal, Karnataka, India

**Keywords:** cell death, chemotherapy, combinatorial therapy, Mito-TEMPO, MTAs, nephrotoxicity

## Abstract

**Background:** The present study reports the protective effect conferred by scavenging mitochondrial oxidative stress (mtOS) in 5-fluorouracil (5-FU)-induced renal injury.

**Methods:** 5-FU renal toxicity model was created by administering 5-FU (12 mg/kg b.w. intraperitoneally [i.p.], for 4 days) to male BALB/c mice. The protective effect of mitochondria-targeted antioxidant (MTA), Mito-TEMPO coadministered at a dosage of 0.1 mg/kg b.w. i.p., was established in terms of levels/expressions of renal injury markers, histopathological alterations, oxidative DNA damage, proinflammatory markers, mtOS, mitochondrial dysfunction, and modulation of apoptotic proteins and apoptotic cell death.

**Results:** A significant rise in the levels of serum urea, uric acid, and creatinine was noted after 5-FU administration to the animals. Immunohistochemical and ELISA findings demonstrated significant decrease in podocin and conversely a significant increase in neutrophil gelatinase-associated lipocalin (NGAL) expression after 5-FU challenge. The histopathological analysis further revealed Bowman's capsule dilation, glomerular condensation, and vacuolar degeneration. Mito-TEMPO treatment significantly lowered renal injury markers, reversed the expressions of podocin and NGAL to normal, and restored normal histoarchitecture of renal tissue. Mitochondrial reactive oxygen species (mtROS), mtLPO, activity of mitochondrial enzyme complexes, and mitochondrial antioxidant defense status were significantly improved in Mito-TEMPO protected group as compared to the 5-FU group. Further, significantly decreased expression of 8-OHdG, reduction in apoptotic cell death, and modulation of apoptotic proteins Bax, Bcl-2, and caspase-3 were noted in Mito-TEMPO protected group, indicating its protective effect against 5-FU-induced renal injury.

**Conclusion:** The approach of targeting mtOS using MTA, Mito-TEMPO, may prove as safe adjuvant in alleviating renal toxicity during 5-FU chemotherapy.

## 1. Introduction

5-Fluorouracil (5-FU) chemotherapy is extensively employed in the treatment of cancers [1]. 5-FU is an antimetabolite chemotherapeutic agent which acts via inhibiting the thymidylate synthase enzyme, thereby creating deficiency of thymine nucleotide. Further, 5-FU gets metabolized into its toxic form, that is, fluorodeoxyuridine monophosphate (F-dUMP), and interferes with nucleic acid synthesis which ultimately leads to the cell death [2]. Although 5-FU holds promising potential in treating various cancers, the notable issue of nonspecific organ toxicity remains a significant concern. This involves major off-target toxicities like cardiotoxicity, renal toxicity, intestinal damage, and hepatotoxicity [3–5]. In recent years, mitochondrial damage emerges as a significant factor linked to 5-FU-induced toxicity, especially in organs with higher mitochondrial density, such as cardiac and renal tissues [6, 7]. Ultrastructural changes in the mitochondria of renal epithelial cells, such as degeneration, elongation, and swelling of mitochondria, are noted after the administration of 5-FU [8]. Moreover, heightened levels of mitochondrial reactive oxygen species (mtROS) and disrupted mitochondrial functioning were also implicated in renal toxicity caused by 5-FU [9–11]. It is believed that 5-FU-induced mitochondrial oxidative stress (mtOS) leads to several major consequences, of which, apoptosis in healthy cells is a major concern [12, 13]. The upregulation of apoptotic proteins such as Bax, Bak, caspase-3, and caspase-9, coupled with the downregulation of antiapoptotic protein Bcl-2, is consistently observed in renal cells following the administration of 5-FU [14–17].

As exceptionally dynamic cellular structures, mitochondria play a pivotal role in a range of functions, including involvement in cellular signaling pathways, the maintenance of redox metabolism, and the generation of ATP [18]. One of the important signaling pathways includes mitochondrial intrinsic apoptotic pathway which reportedly gets activated upon excessive mtOS generation [19–22]. Increased levels of oxidative stress within the mitochondria lead to a reduction in the inner mitochondrial membrane potential (*ΔΨ*m), culminating in the initiation of the mitochondrial permeability transition pore opening localized in the inner mitochondrial membrane and perforations in the outer mitochondrial membrane facilitated by proteins from the Bcl-2 family [21, 23, 24]. Under normal conditions, a balance is maintained between proapoptotic and antiapoptotic Bcl-2 family proteins. However, upon activation, proapoptotic proteins form pores in the outer mitochondrial membrane, leading to mitochondrial membrane disruption and the release of cytochrome c. This event ultimately triggers caspase-mediated apoptotic cell death [25]. Multiple in vivo studies confirmed 5-FU-induced oxidative damage to mitochondria such as depolarization of mitochondrial membrane and mitochondrial swelling led apoptotic cell death of myocardium [12, 26]. Our previous reports were also in concurrence with these findings where we reported significantly raised levels of mtOS and increased apoptotic cell death after 5-FU administration in mice [27, 28]. This underscores the necessity for an agent that can specifically target mtOS and prevent further downstream consequences within normal cells during chemotherapy.

In the recent years progress has been made to target drugs/antioxidants specifically to the mitochondria by conjugating them with lipophilic cations such as triphenylphosphonium. The higher mitochondrial membrane potential (150–180 mV as compared to 70–90 mV of cell membrane) drives the accumulation of conjugated antioxidant (mitochondria-targeted antioxidant [MTA]) 100- to 1000-folds in mitochondrial matrix and specifically decreases the mtROS [29]. Mito-TEMPO is one of the MTAs which effectively decreases mtOS [30, 31]. It has shown its significant protective effect against a wide variety of oxidative stress-related diseases such as cardiovascular diseases [32], renal fibrosis [33], hepatic disorders [34], neurodegenerative disorders [35], and cancers [36–38]. Considering this, in the present study, we explored the protective effect of Mito-TEMPO against 5-FU-induced renal injury.

## 2. Material and Methods

### 2.1. Chemicals and Reagents

5-FU, Mito-TEMPO, thiobarbitric acid, pyrogallol, 5,5′-dithiobis-2-nitrobenzoic acid, and rhodamine-123 were procured from Sigma–Aldrich, USA. Antibodies used to detect inflammatory markers including IL-6 (bs0782R), IL-10 (PA5-85,660), TNF-α (bs-2081R), apoptotic markers Bax (MA5-14003), Bcl-2 (MA5-11757), caspase-3 (MA1-91637), and oxidative DNA damage marker 8-OHdG (bs-1278R), as well as the goat anti-rabbit IgG secondary antibody (65–6120), were obtained from Thermo Fisher Scientific, Inc., Rockford, IL, USA. In situ Apoptosis Detection Kit, TACS-XL, was procured from Trivigen, Japan. The remaining chemicals and reagents utilized in the study were sourced from local Indian firms and were of the highest purity grade.

### 2.2. Experimental Treatment and Animal Grouping

**Figure 1:**
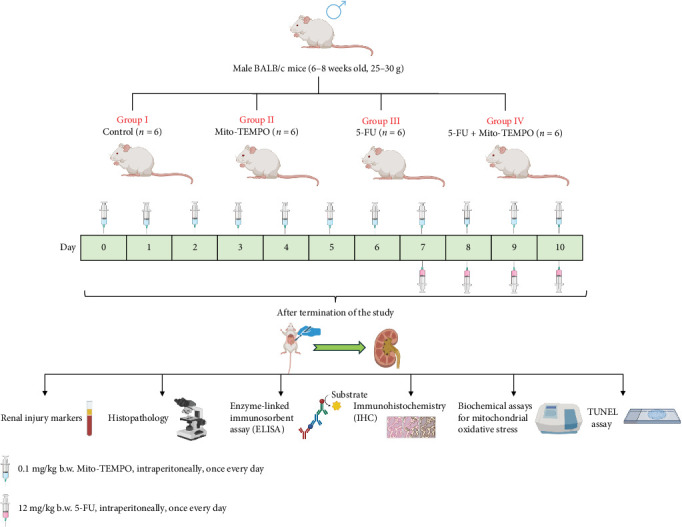
Diagrammatic representation of animal grouping, drug dosing, and experimental parameters.

Institutional Animal Ethics Committee approval (IAEC/KMC/54/2021) was sought to carry out animal experiments. Healthy male BALB/c mice in a weight range of 25–30 g and aged between 6 and 8 weeks were housed in controlled environmental conditions. The Committee for the Purpose of Control and Supervision of Experiments on Animals (CPCSEA) guidelines (regulatory guidelines for handling of experimental animals, Government of India) were strictly followed for handling of the animals. Upon the completion of acclimatization period of 7 days, all animals were randomly segregated into four groups (*n* = 6 in each group). 5-FU was administered to the animals of 5-FU group at a dose of 12 mg/Kg b.w. intraperitoneally (i.p.) for consecutive 4 days [9, 39]. Mito-TEMPO was administered to the animals of Mito-TEMPO group at a dose of 0.1 mg/kg b.w. i.p. once every day till the completion of the experiment [37, 38]. 5-FU + Mito-TEMPO group received 5-FU and Mito-TEMPO as described for the mentioned groups. Mito-TEMPO treatment initiated 2 weeks prior to the 5-FU treatment (Figure 1). Upon completion of dose regimen, blood samples were collected, and animals were sacrificed by cervical dislocation to obtain kidney tissue samples for further assessments.

### 2.3. Renal Injury Markers

Blood samples were obtained from retro-orbital plexus of mice, and serum was separated using standard laboratory protocols. Serum samples obtained from different groups of animals were further used for the estimation of the levels of renal injury markers: serum urea, serum uric acid, and serum creatinine using standard commercial kit (Liqui check, Agappe Diagnostics, India). In addition, the expression of renal injury markers like podocin and neutrophil gelatinase-associated lipocalin (NGAL) was assessed using immunohistochemistry technique, and the levels of these markers were further confirmed using ELISA.

### 2.4. Histopathological Assessment of Renal Tissue

Histoarchitectural alterations in the renal tissue of mice from different treatment groups were assessed using Hematoxylin and eosin (H&E) staining. Renal tissues were fixed in formalin (10% w/v) for a period of 24 h followed by dehydration subsequent embedding in paraffin wax. Further, tissue sections of 5 μm thickness were affixed onto clean slides (precoated with albumin). For staining, these slides were deparaffinized and rehydrated using descending concentrations of alcohol and stained with hematoxylin solution for 10 min. The stained slides were rinsed in distilled water to remove excess hematoxylin. Subsequently, slides were immersed in bluing reagent (ammonia water) for 2 min and then rinsed in distilled water. Lastly, slides were stained in eosin solution and embedded in dibutylphthalate polystyrene xylene (DPX) for observation under a light microscope (Labomed, Lx 300, USA) [40].

### 2.5. ELISA of Inflammatory Markers and Apoptotic Markers in Renal Tissue

In order to assess the expressions of renal inflammatory cytokines (such as IL-6, IL-10, and TNF-α) and the expression of renal apoptotic markers (such as Bax, Bcl-2, and caspase-3), ELISA was performed. Briefly, antigens for the respective markers from kidney tissues were extracted using antigen extraction buffer (Tris-HCl–sodium deoxycholate–TritonX–EDTA; pH7.4) as described earlier [27]. Flat bottom 96-well polystyrene ELISA plates (Himedia, India) were used to incubate the extracted antigens for 12 h at 4°C. Upon the completion of incubation period, the solution was drained, and plates were washed with wash buffer (PBSTw) and blocked with blocking solution (1% bovine serum albumin [BSA]). Further, 100 μL of primary antibodies of IL-6 (bs0782R), IL-10 (PA5-85,660), TNF-α (bs-2081R), Bax (MA5-14003), Bcl-2 (MA5-11757), and caspase-3 (MA1-91637) were added to the corresponding wells. The plates were further kept for incubation at room temperature for 2 h in order to facilitate antigen–antibody interaction. Upon completion of incubation period, the plates were rinsed with wash bugger, and 100 μL of goat antirabbit IgG secondary antibody was added to each well. Finally, plates were spectrophotometrically read in ELISA multiplate reader at wavelength of 420 nm (Lisa Plus, Rapid diagnostics, India).

### 2.6. Immunohistochemistry of Oxidative DNA Damage Marker and Apoptotic Proteins in Renal Tissue

Immunohistochemical analysis for the oxidative stress marker 8-OHdG and apoptotic markers Bax, Bcl-2, and caspase-3 was carried out according to the previously described method [38]. Briefly, paraffin-embedded kidney tissue sections were deparaffinized by prewarming slides in oven at 55°C and passing through multiple changes of xylene. Deparaffinized tissue slides were further rehydrated using descending concentrations of alcohol. Antigen retrieval from the rehydrated tissue was carried out by immersing the slides in a 10 mM sodium citrate solution (pH 6.0), followed by a 20-min treatment with 1% H_2_O_2_ at room temperature. To block nonspecific binding, 10% BSA was used, and the slides were incubated for 1 h at room temperature. After washing with PBS, the slides were incubated with primary antibodies for 8-OHdG, Bax, Bcl-2, and caspase-3 for 2 h at room temperature. Subsequently, the slides were incubated with a horseradish peroxidase (HRP)-labeled secondary antibody for 1 h at room temperature. Finally, the slides were treated with 1% 3,3′-diaminobenzidine (DAB), dehydrated using ascending concentrations of alcohol, and mounted using DPX for observation under a light microscope (Labomed, Lx 300, USA)

### 2.7. Cell Death Analysis (TUNEL) in Renal Tissue

Immunohistochemical staining of kidney tissues was performed to evaluate cell death using the TUNEL assay technique, using a commercially available kit (TACSXL In Situ Apoptosis Detection Kit, Trivigen, Japan). Briefly, deparaffinized and rehydrated tissue sections were allowed to incubate with proteinase K at 37 °C for 15 min. Following this, slides were rinsed with deionized water for 2 min and immersed in a quenching solution (30% H_2_O_2_ in methanol) for 5 min. After PBS wash, slides were immersed in 1× terminal deoxynucleotidyl transferase (TdT) labeling buffer and incubated with a biotin-deoxyribonucleotide triphosphate (B-dNTP) labeling reaction mixture in a humidification chamber at 37°C for a duration of 30 min. After treatment with stop buffer, slides were incubated with an anti-bromodeoxyuridine (anti-BrdU) antibody solution within a humidification chamber at 37°C for a period of 30 min. Following the washing with PBS Tween 20, slides were immersed in a streptavidin–HRP (Strep-HRP) solution and counterstained using methyl green for visualization in light microscopy (Lx 300, Labomed, USA). The apoptotic index was calculated as percentage of number of apoptotic cells identified to the total number of cells.

### 2.8. mtOS and Mitochondrial Enzymatic Activity in Renal Tissue

mtOS was evaluated by isolating mitochondria from kidney tissues, following earlier protocol [41]. Renal tissue was homogenized in ice-cold buffer (0.25 M sucrose, 10 mM Tris-HCl, 0.5 mM EDTA; pH 7.4) and centrifuged at 2100× *g* for 15 min at 4°C. The supernatant was further centrifuged at 14,000× *g* for 20 min at 4°C, and the resulting mitochondrial pellet was resuspended in suspension buffer (0.44 M sucrose, 10 mM Tris-HCl; pH 7.4). mtROS and mtLPO were measured as previously described [42, 43]. For mtROS assessment, the mitochondrial fraction was incubated with DCFH-DA-methanol solution (1.25 mM) at 37°C for 30 min, and changes were noted using a fluorimeter (FP8300, Jasco, USA) at excitation and emission wavelengths of 500 nm and 520 nm, respectively. For mtLPO, the mitochondrial fraction was treated with TCA–TBA–HCl solution and centrifuged at 1000× *g* for 10 min. The resulting changes in mtLPO were measured spectrophotometrically at 532 nm and expressed as nmol MDA/mg mitochondrial protein.

### 2.9. Activity of Mitochondrial Complexes, TCA Cycle Enzymes, and Mitochondrial Antioxidant Enzymes in Kidney Tissue

The activities of mitochondrial complexes I, II, and IV were determined using previously established protocols [44, 45]. For complex I activity, the reaction mixture, consisting of 0.03 M potassium ferricyanide, was incubated at 30°C, followed by the addition of 1% NADH and the mitochondrial fraction. The change in absorbance at 420 nm was measured for 3 min, and the activity was expressed as nmol of NADH oxidized/min/mg of mitochondrial protein. Complex II activity was assessed by adding a reaction mixture containing 0.6 M succinic acid and 1% BSA to the mitochondrial suspension. The absorbance change at 420 nm was recorded for 3 min, and the activity was expressed as nmol of SDH/min/mg of mitochondrial protein. Complex IV activity was measured according to Pearl et al. [45]. Briefly, the mitochondrial suspension was incubated at 25°C for 5 min, followed by the addition of the reaction mixture (0.2% N-phenyl-p-phenylenediamine, 0.01% cytochrome C in 0.03 M phosphate buffer, pH 7.4), and absorbance at 550 nm was recorded for 3 min. Enzyme activity was expressed as nmol of cytochrome c oxidized/min/mg of mitochondrial protein [45]. Additionally, the activities of MDH and IDH were determined according to previously described methods [46, 47]. The activities of mitochondrial antioxidant enzymes, including mtGSH [48], mtGR [49], mtGPx [46], and MnSOD [50], were evaluated using standard biochemical methods.

### 2.10. Estimation of Mitochondrial Membrane Potential

Mitochondrial membrane potential was evaluated using the method outlined by previous studies [51]. In brief, the mitochondrial fraction was incubated with a reaction mixture consisting of 150 mM sucrose, 4 mM MgCl2, 30 mM HEPES-KOH, and 5 mM K_2_HPO_4_ (pH 7.4) at 37°C for 5 min. The reaction was initiated by the addition of 5 μM rhodamine 123, and the fluorescence of the samples was measured at an excitation wavelength of 507 nm and emission wavelength of 527 nm using a fluorimeter (FP8300, Jasco, USA).

### 2.11. Protein Estimation

The protein concentration in mitochondrial fractions was estimated using Lowry's method [52]. BSA was kept as protein standard, and optical density of samples was recorded at 620 nm spectrophotometrically.

### 2.12. Statistical Analysis

The normality of the data was assessed using the Shapiro–Wilk test, and the homogeneity of variance was evaluated with Levene's test. Intergroup comparisons for different parameters were performed using one-way analysis of variance (ANOVA), followed by Tukey's honestly significant difference (HSD) post hoc test. A *p*-value of ≤0.05 was considered statistically significant.

## 3. Results

### 3.1. Mito-TEMPO Treatment Prevented 5-FU-Induced Renal Damage

**Figure 2:**
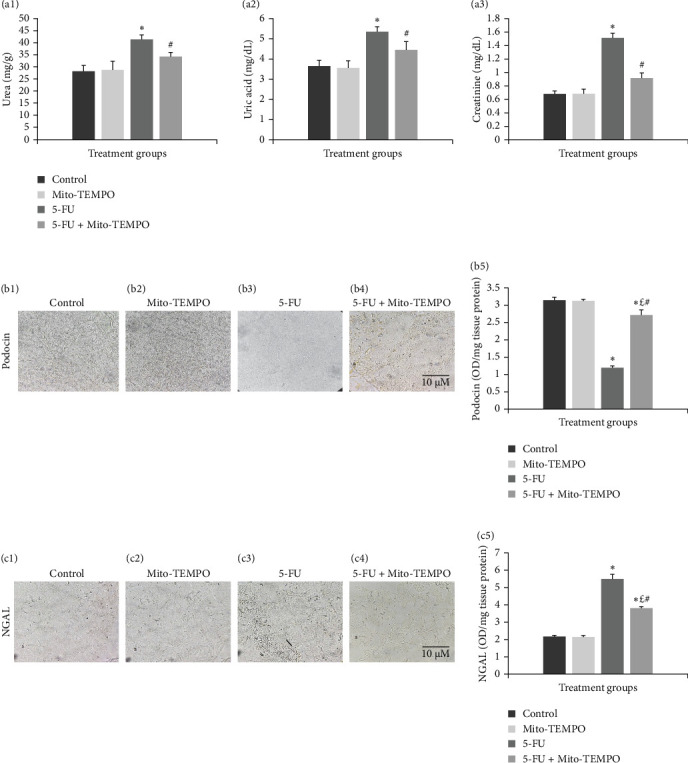
Protective effect of Mito-TEMPO against 5-FU-induced renal injury. (A) Serum levels of renal injury markers: (a1) the effect of Mito-TEMPO on the levels of serum urea in different treatment groups. (a2) The effect of Mito-TEMPO on the levels of serum uric acid in different treatment groups. (a3) The effect of Mito-TEMPO on the levels of serum creatinine in different treatment groups. (B) Expression of podocin using immunohistochemistry and ELISA: (b1) control and (b2) Mito-TEMPO group showing darkly stained, brown-colored positive immunostaining for podocin protein in renal tissue. (b3) 5-FU group showing decreased expression of podocin. (b4) 5-FU + Mito-TEMPO group showing restored expression of podocin. (b5) Graph showing ELISA results for podocin expression in different treatment groups. (C) Expression of NGAL using immunohistochemistry and ELISA: (c1) control and (c2) Mito-TEMPO group showing uniformly light stained renal tissue. (c3) 5-FU group showing positive immunostaining indicated by intense dark brown stained cells. (c4) 5-FU + Mito-TEMPO group showing decreased brown color immunostaining intensity. (c5) Graph showing ELISA results for NGAL expression in different treatment groups (data were expressed as mean ± SD and analyzed using one-way ANOVA followed by post hoc test [Tukey's HSD]. *⁣*^*∗*^ represents *p* ≤ 0.05 when compared with the control group. £ represents *p* ≤ 0.05 when compared with the Mito-TEMPO group. # represents *p* ≤ 0.05 when compared with the 5-FU group).

The administration of 5-FU to mice at a dose of 12 mg/kg b.w. resulted in notable change in renal health which was assessed in terms of renal injury markers. 5-FU challenged animals demonstrated significantly increased (*p* ≤ 0.05) serum levels of kidney injury markers like urea, uric acid, and creatinine when compared to the control group animals. Mito-TEMPO treatment prevented the renal injury as demonstrated by significantly decreased (*p* ≤ 0.05) serum levels of urea (1.20-fold), uric acid (1.23-fold), and creatinine (1.66-fold) in 5-FU + Mito-TEMPO group animals (Figure 2A (a1–a3). A similar protection was also observed when evaluated using podocin and NGAL kidney injury tissue markers. Podocin and NGAL are considered novel renal injury markers which are highly sensitive to renal damage. The expression of podocin in healthy animals (control group) and drug control group (Mito-TEMPO group) animals were immunohistochemically confirmed and characterized as darkly stained, brown coloration within renal tissue. The toxicity group (5-FU group) animals had decreased intensity of immunostaining for podocin within renal tissue when compared to healthy (control group) animals. Treatment of Mito-TEMPO to 5-FU-challenged animals (5-FU + Mito-TEMPO group) notably increased the expression of podocin as indicated by increased brown coloration when compared to untreated toxicity group (5-FU group) animals, indicating its protective potential. These alterations were further supported by ELISA findings where toxicity group (5-FU group) animals showed a significant (*p* ≤ 0.05) drop (2.50-fold) in the levels of podocin in comparison to healthy (control group) animals. The treatment of Mito-TEMPO to 5-FU-challenged animals (5-FU + Mito-TEMPO group) significantly (*p* ≤ 0.05) raised the levels of podocin by 2.25-fold in comparison to untreated toxicity group (5-FU group) animals (Figure 2B (b1–b5)). Additionally, immunostaining for NGAL demonstrated increased expression of NGAL in toxicity group (5-FU group) animals in comparison to healthy (control group) animals. Treatment with Mito-TEMPO to toxicity group animals notably decreased the intensity of NGAL immunostaining in 5-FU + Mito-TEMPO group in comparison to 5-FU group animals. These alterations in the expression of NGAL were also supported with ELISA findings where toxicity group (5-FU group) animals demonstrated significantly (*p* ≤ 0.05) raised levels of NGAL (2.52-fold) in comparison to healthy group (control group) animals. The prevention of renal injury by Mito-TEMPO was clearly evident in treatment group (5-FU + Mito-TEMPO group) animals as they showed significantly (*p* ≤ 0.05) decreased level of NGAL (1.44-fold) in comparison to toxicity group (5-FU group) animals (Figure 2C (c1–c5)).

**Figure 3:**
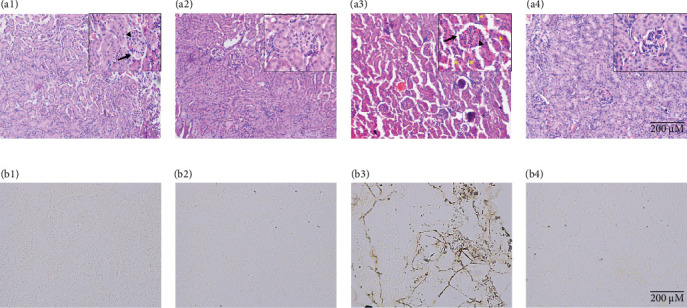
(A) Protective effect of Mito-TEMPO on histoarchitecture of different treatment groups: (a1 and a2) control group and Mito-TEMPO group showing normal renal tubules, glomerulus (arrowhead), and Bowman's capsule (arrow), respectively. (a3) 5-FU-challenged group demonstrating dilation of Bowman's capsule (arrow), glomerular congestion/condensation (arrowhead), and overall vacuolar degeneration (yellow asterisk). (a4) 5-FU + Mito-TEMPO group showing restored/protected histoarchitecture of renal tissue. (B) Protective effect of Mito-TEMPO on oxidative DNA damage in various treatment groups: (b1) control group and (b2) Mito-TEMPO group showing normal renal cells without any intense coloration in the tissue. (b3) 5-FU group showing brown and darkly stained renal cells. (b4) 5-FU + Mito-TEMPO group showed light stained 8-OHdG immunostaining in renal tissue.

Further, histopathological assessment of H&E-stained renal tissue revealed that healthy (control) and drug control (Mito-TEMPO) group animals exhibited normal tissue architecture with clearly visible renal tubules, Bowman's capsule, and glomerulus (Figure 3A (a1,a2)). 5-FU-challenged, that is, toxicity group animals demonstrated noticeable histological changes such as dilation of Bowman's capsule, glomerular congestion/condensation, and overall vacuolar degeneration (Figure 3A (a3)). However, Mito-TEMPO treatment to 5-FU-challenged animals successfully prevented the alterations in histoarchitecture of renal tissue in treatment group (5-FU + Mito-TEMPO group) animals in comparison to toxicity group animals (Figure 3A (a4)).

Furthermore, oxidative DNA damage in renal tissue was confirmed by 8-OHdG immunostaining. Healthy group (control group) and drug control group (Mito-TEMPO group) animals demonstrated normal renal tubules without any dark coloration of tissue (Figure 3B (b1,b2)). However, brown and intensely stained renal tubules were observed in toxicity group (5-FU group) animals, confirming increased presence of 8-OHdG in comparison to healthy group (control group) animals (Figure 3B (b3)). Mito-TEMPO treatment to 5-FU-challenged animals remarkably decreased the intensity of 8-OHdG immunostaining in treatment group (5-FU + Mito-TEMPO group) animals in comparison to toxicity group (5-FU group) animals (Figure 3B (b4)), clearly demonstrating its preventive action against 5-FU-induced renal oxidative damage.

### 3.2. Mito-TEMPO Ameliorated 5-FU-Induced mtOS and Restored the Activities of Mitochondrial Enzymes

5-FU challenge significantly (*p* ≤ 0.05) raised the levels of mtLPO and mtROS in toxicity group (5-FU group) animals in comparison to healthy group (control group) animals. However, pretreatment with Mito-TEMPO to 5-FU-challenged animals significantly (*p* ≤ 0.05) decreased the levels of mtLPO and mtROS in treatment group (5-FU + Mito-TEMPO group) animals demonstrating 1.10 and 1.22-fold decrease in comparison to toxicity group (5-FU group) animals, respectively. Further, 5-FU-challenge significantly (*p* ≤ 0.05) decreased the mitochondrial membrane potential by 1.40-folds in toxicity group (5-FU group) animals in comparison to healthy group (control group) animals. Remarkably, the administration of Mito-TEMPO to animals challenged with 5-FU resulted in a significant (*p* ≤ 0.05) increase in mitochondrial membrane potential, with a 1.23-fold augmentation observed in the treatment group (5-FU + Mito-TEMPO group) animals in comparison to the toxicity group (5-FU group) animals (Table 1).

Given the possibility that mtOS could have increased due to impaired mitochondrial functioning, we further investigated the activities of mitochondrial complexes (complex I, II, and IV) and TCA cycle enzymes: IDH and MDH. As expected, a significant impairment (*p* ≤ 0.05) in the activities of mitochondrial respiratory chain complexes (like complex I, complex II, and complex IV) and TCA cycle enzymes (such as IDH and MDH) were noted in toxicity group (5-FU group) animals in comparison to healthy (control group) animals. However, pretreatment with Mito-TEMPO significantly (*p* ≤ 0.05) improved the enzymatic activities of mitochondrial respiratory chain complexes as well as IDH and MDH enzymes in treatment group (5-FU + Mito-TEMPO group) animals in comparison to the toxicity group (5-FU group) animals (Table 1).

Additionally, to underline the role of oxidative stress in 5-FU-induced renal damage, we conducted assessment of mitochondrial antioxidant enzyme activities which generally play a pivotal role in maintaining redox homeostasis and helps in scavenging of free radicals. Expectedly, toxicity group (5-FU group) animals demonstrated significant (*p* ≤ 0.05) drop in the activities of mtGSH, mtGR, mtGPx, and mtSOD by 1.84-, 2.05-, 1.89-, and 2.42-folds, respectively, in comparison to the healthy group (control group) animals. However, pretreatment with Mito-TEMPO in animals challenged with 5-FU exhibited a significant (*p* ≤ 0.05) enhancement in the enzymatic activities of mtGSH, mtGR, mtGPx, and mtSOD by 1.39-, 1.55-, 1.53-, and 1.85-folds increase respectively in treatment group (5-FU + Mito-TEMPO group) animals in comparison to toxicity group (5-FU group) animals (Table 1).

### 3.3. Mito-TEMPO Treatment Alleviated the Apoptotic Cell Death

**Figure 4:**
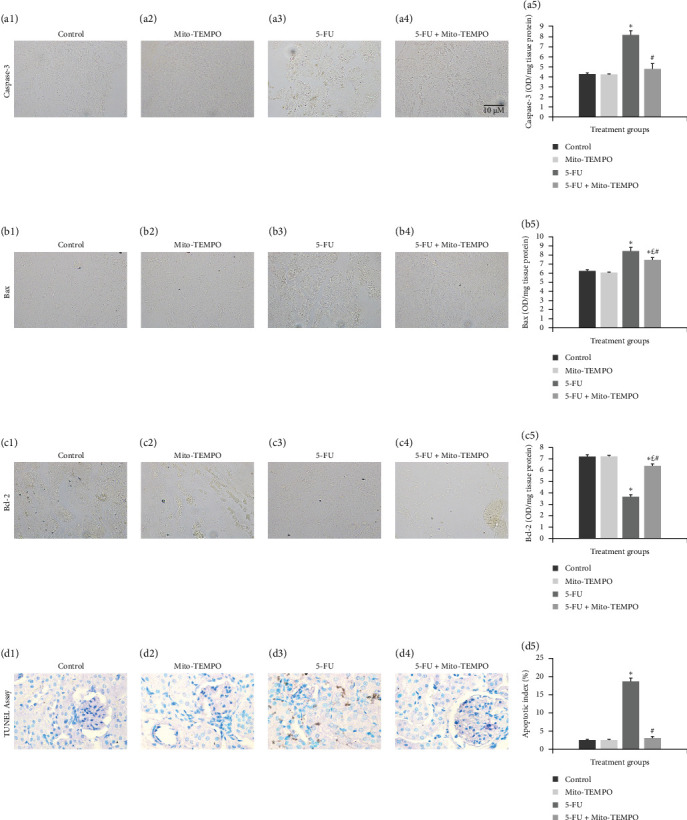
Protective effect of Mito-TEMPO against 5-FU-induced apoptosis in renal tissue. (A) Expression of caspase-3 proteins in different treatment groups: (a1 and a2) control and Mito-TEMPO group immunohistochemistry showing normal renal tubules with uniformly light stained cells. (a3) 5-FU group showing intense immunostaining indicated by dark brown stained cells. (a4) 5-FU + Mito-TEMPO group showing relatively decreased intensity of immunostaining as compared to the 5-FU group. (a5) Graph showing ELISA results for the expression of caspase-3 in different treatment groups. (B) Expression of Bax proteins in different treatment groups: (b1 and b2) control and Mito-TEMPO group showing normal, uniformly stained renal tissue. (b3) 5-FU group demonstrating increased intensity of immunostaining indicated by dark brown stained cells. (b4) 5-FU + Mito-TEMPO group showing relatively decreased intensity of immunostaining as compared to the 5-FU group. (b5) Graph showing ELISA results for the expression of Bax proteins in different treatment groups. (C) Expression of Bcl-2 proteins in different treatment groups: (c1 and c2) control and Mito-TEMPO group showing intense immunostaining indicated by darkly stained brown renal tissue. (c3) 5-FU group demonstrating decreased intensity of immunostaining. (c4) 5-FU + Mito-TEMPO group showing relatively increased intensity of immunostaining as compared to 5-FU group. (c5) Graph showing ELISA results for the expression of Bcl-2 proteins in different treatment groups. (D) Protective effect of Mito-TEMPO against apoptotic cell death: (d1) control group and (d2) Mito-TEMPO group showing normal renal cells with distinct blue stained nuclei and with no visible TUNEL-positive cells. (d3) 5-FU group demonstrating dark brown stained nuclei indicating TUNEL-positive cells. (d4) 5-FU + Mito-TEMPO showing very few TUNEL-positive cells. (d5) Graph showing percent apoptotic index in different treatment groups. (Data were expressed as mean ± SD and analyzed using one-way ANOVA followed by post hoc test [Tukey's HSD]. *⁣*^*∗*^ represents *p* ≤ 0.05 when compared with the control group. £ represents *p* ≤ 0.05 when compared with the Mito-TEMPO group. # represents *p* ≤ 0.05 when compared with the 5-FU group).

The mtOS can lead to apoptosis via the modulation of intrinsic mitochondrial apoptotic pathway. The immunohistochemical analysis clearly showed that toxicity group (5-FU group) animals had increased intensity of immunostaining for caspase-3 and Bax protein as indicated by darkly stained, brown coloration within renal tissue in comparison to healthy group (control group) animals (Figure 4A (a3) and Figure 4B (b3)). However, Mito-TEMPO administration to 5-FU- challenged animals demonstrated notable decrease in the positively stained renal tubules in treatment group (5-FU + Mito-TEMPO group) animals in comparison to toxicity group (5-FU group) animals (Figure 4A (a4) and Figure 4B (b4)). Further, antiapoptotic cytokine immunostaining was decreased in toxicity group (5-FU group) animals in comparison to the heathy group (control) and treatment group (i.e., 5-FU + Mito-TEMPO) animals (Figure 4C (c3)). The immunohistochemistry observations were further supported by ELISA readings for the expressions of these apoptotic and antiapoptotic cytokines. The toxicity group (5-FU group) animals demonstrated significantly (*p* ≤ 0.05) raised levels of caspase-3 and Bax proteins in comparison to healthy group (control group) animals. Mito-TEMPO administration in treatment group (5-FU + Mito-TEMPO group) animals significantly (*p* ≤ 0.05) decreased the levels of caspase-3 and Bax proteins in comparison to toxicity group (5-FU group) animals (Figure 4A (a5) and Figure 4B (b5)). Expectedly, toxicity group (5-FU group) animals further showed significant decrease in the levels of Bcl2 proteins in comparison to healthy group (control group) animals. However, treatment group animals (in 5-FU + Mito-TEMPO group) demonstrated significantly raised levels of Bcl2 proteins in comparison to toxicity group (5-FU group) animals (Figure 4C (c5)). Finally, TUNEL assay was carried out to assess the manifestation of action of these cytokines in terms of apoptotic cell death. Healthy group (control) and drug control group (Mito-TEMPO group) animals showed very few apoptotic cells with apoptotic index (%) 2.41 ± 0.236 and 2.40 ± 0.228, respectively (Figure 4D (d1,d2,d5)). The toxicity group (5-FU group) animals demonstrated darkly stained, brown TUNEL-positive apoptotic cells with significantly (*p* ≤ 0.05) increased apoptotic index (%) 18.58 ± 0.913 in comparison to healthy group (control group) animals (Figure 4D (d3,d5)). The treatment group animals (5-FU + Mito-TEMPO group) showed very few TUNEL-positive cells and significantly (*p* ≤ 0.05) normalized the apoptotic index (%) 2.93 ± 0.466 that demonstrates Mito-TEMPO successfully prevented renal cells from 5-FU-induced apoptotic cell death (Figure 4D (d4,d5))

## 4. Discussion

5-FU is an effective chemodrug but also exerts significant nonselective toxicity in vital organs such as the heart, intestines, kidneys, and liver [9]. Numerous approaches have been applied to mitigate the associated toxicity amongst which plasma level dose adjustment, synergistic formulations including prodrugs, and combination therapy are the most common strategies [53, 54]. Despite various efforts, still 5-FU toxicity remains one of the major concerns. For example, even the recent approach of using derivatives/prodrugs of 5-FU which is known to reduce off-target toxicity is also associated with toxic side effects such as cardiotoxicity, nephrotoxicity, neutropenia, stomatitis, diarrhea, neurotoxicity, oxidative stress, and mitochondrial dysfunction [26, 55–57]. Considering the well-established association between mitochondrial dysfunction and chemotherapy-induced nephrotoxicity [58–60], we speculated that protecting mitochondria of normal cells during 5-FU administration could be a promising approach to overcome 5-FU-induced renal toxicity.

5-FU-induced nephrotoxicity in vivo model was developed by administrating 5-FU at the dosage of 12 mg/kg b.w. i.p. daily. In present study, renal injury was confirmed by significant alterations in the levels/expression of kidney injury serum markers as well as renal tissue injury markers podocin and NGAL. Podocin and NGAL are highly sensitive kidney injury marker. Podocin is a crucial protein present predominantly in the slit diaphragm of podocytes in the kidney's glomerular filtration barrier, and any damage to podocytes potentially leads to the disruption of slit diaphragm and release of podocin into the urine [61]. NGAL in contrast is released by neutrophils in response to inflammation [62]. Diseased conditions such as proteinuria renal disease and diabetic nephropathy are known to decrease the renal tissue expression of podocin [63, 64]. In a study conducted by Al-Ghamdi et al. [65], exposure to 5-FU significantly increased serum levels of NGAL. Our immunohistochemical and ELISA findings corroborate with these results and showed a significant decreased expression of podocin and alternatively a significantly increased expression of NGAL in toxicity group (5-FU group) animals in comparison to healthy group animals.

The renal injury was further supported by histopathological findings where 5-FU treatment demonstrated glomerular congestion, dilation of Bowman's capsule, and vacuolar degeneration of renal tubules. These results were in corroboration with previous in vivo studies where 5-FU treatment demonstrated similar morphological changes in renal tissue [11, 17]. Glomerular congestion by heightened intraglomerular pressure through renal vasoconstriction and tubular damage is observed in case of several chemotherapeutic drugs [66, 67]. Several animal studies demonstrated 5-FU-induced glomerular congestion as one of the major glomerular lesions [3, 68]. The engorgement (bulging) of glomerular capillaries exerts pressure on the surrounding structures, leading to podocyte injury [69]. Consequently, there will be increased filtration of proteins and macromolecules, contributing to fluid accumulation and the dilation of Bowman's capsule [70]. Several ultrastructural in vivo investigations confirmed the 5-FU-induced distortion and destruction of foot processes of the podocytes [8, 11]. In the present findings, the decreased podocin expression in 5-FU-challenged animals correlates well with the abovementioned histological changes. The exact mechanism by which 5-FU induces nephrotoxicity remains unclear; however, a widely postulated mechanism involves the production of free radicals, lipid peroxidation, cell membrane components damage, lysosomal enzyme activation, and apoptotic cell death [71, 72]. Although there is limited literature on the specific role of mtOS in 5-FU-induced nephrotoxicity, it is well established that elevated mtOS activates various kinase pathways in mesangial cells, the structural units of the glomerulus, resulting in alterations to podocytes and glomerular proteins [73]. This process activates profibrotic pathways, contributes to glomerulosclerosis, and disrupts the glomerular filtration barrier, ultimately resulting in proteinuria [74]. The present study's observations that Mito-TEMPO pretreatment successfully ameliorated these damaging effects of 5-FU also corroborated with another in vivo study which revealed that inadequate levels of mitochondrial superoxide dismutase (SOD2/MnSOD) worsened renal pathologies and accelerated ROS-induced tubular damage, renal cellular senescence, renal interstitial inflammation, and glomerular sclerosis [75].

Mitochondria serve as the principal site for the generation of ROS, and the electron transport chain (ETC) complexes act as a predominant source of these free radicals [76]. The electrons leaking from the ETC exhibits univalent reduction of molecular oxygen leading to generation of ROS within mitochondria. The resulting superoxide radical, characterized by its high reactivity, reacts with macromolecules in close proximity resulting in mitochondrial dysfunction and apoptosis [77]. Mito-TEMPO is a MTA which is composed of a lipophilic TPP^+^ group and an antioxidant moiety, piperidine nitroxide (TEMPO) [78]. The lipophilic cation TPP^+^ facilitates a five- to tenfold accumulation of antioxidants into the cytoplasm across the plasma membrane, which has a potential of approximately −60 mV [79]. The mitochondrial membrane, with a higher potential of −150 to 180 mV, further drives the accumulation of antioxidants into the mitochondria, resulting in an overall increase of 100- to 1000-fold relative to the extracellular compartment [79]. TEMPO, being a superoxide dismutase mimetic, specifically facilitate dismutation of mitochondrial superoxide ions [80]. It is further supported by our observation where treatment of Mito-TEMPO resulted in a substantial reduction in the levels of mtROS. This protective effect was also observed in terms of improvement in the mitochondrial antioxidant defense system as indicated by the improvement in the activities of mitochondrial respiratory chain complexes and mitochondrial antioxidant defense enzymes. To further verify our findings, we investigated the status of 8-OHdG in renal tissue. 8-OHdG is an important marker for intrarenal oxidative stress caused by 5-FU [81]. It represents one of the prevalent forms of oxidative lesions resulting from free radicals in both nuclear and mitochondrial DNA [82, 83]. The significant lowering of 8-OHdG in 5-FU-challenged kidney cells in Mito-TEMPO-protected group (treatment group) clearly indicated protective effect of Mito-TEMPO during 5-FU challenge to the animals. Our results were consistent with findings of other research groups where Mito-TEMPO was shown to mitigate the podocyte injury and loss of podocytes in oxidative stress-related kidney diseases [33, 84–87].

Further, mitochondrial ROS is a major determinant of cell apoptosis in normal cells [88]. As mentioned earlier, the present study has already confirmed heightened mtOS in the 5-FU group, and the significant augmentation in the expression of proapoptotic proteins and the apoptotic index in toxicity group (5-FU group) animals might indicate the role of mtROS in inducing 5-FU-mediated apoptosis. The elevated mtOS can initiate the mitochondria-mediated intrinsic apoptosis pathway, resulting in the release of apoptosis-inducing factors, such as cytochrome c, from the intermembrane space (IMS) into the cytosol [21, 89]. It has been proposed that multiple pathways and mechanisms may coexist in the release of cytochrome c, with mitochondrial ROS playing a role in influencing the opening of the permeability transition pore, the voltage-dependent anion channel, inner membrane proteins such as ANT, and BH3 proteins [90–92]. The direct effect of mitochondrial ROS causes oxidation of thiol groups of inner membrane proteins like ANT, which is also an important component of PTP. Following its release, cytochrome c associates with Apaf-1 and procaspase-9, establishing a multiprotein complex recognized as the apoptosome. This interaction ultimately activates caspase-9, which in turn activates the executioner protein caspase-3 leading to cell death [93]. In case of 5-FU, multiple studies demonstrated that administration of 5-FU led to the apoptosis of renal cells via activation of intrinsic mitochondrial apoptotic pathway [14–17].

Collectively, these findings indicated that mtOS is an important contributor of renal injury induced by 5-FU, and the approach of targeting mtOS using MTAs is promising in mitigating renal toxicity during chemotherapy. The kidney's high metabolic demands necessitate robust mitochondrial function for ATP production, redox balance, apoptosis regulation, and calcium homeostasis. Injury to renal mitochondria disrupts these processes, leading to oxidative stress, reduced ATP production, and apoptotic cell death, contributing to conditions like chronic kidney disease (CKD) [94]. In the present study, Mito-TEMPO has alleviated renal injury by scavenging mitochondrial ROS, thereby likely mitigating downstream effects like impaired electron transfer chain activities, drop in mitochondrial membrane potential, oxidative damage to DNA, and finally mitochondria-mediated intrinsic apoptotic renal cell death.

## 5. Conclusion

The findings of the current study provide additional insights into the mechanisms contributing to 5-FU-induced renal toxicity. The implications of these results hold potential clinical significance, particularly in the context of combinatorial therapy as Mito-TEMPO can be used as an adjuvant with the conventional chemotherapy regimen. However, further confirmatory studies are required both at preclinical and clinical levels to validate and establish that the administration of Mito-TEMPO does not compromise the anticancer efficacy of the chemotherapeutic drug and, consequently, the overall therapeutic outcome of the regimen.

## Figures and Tables

**Table 1:** Effect of Mito-TEMPO on mitochondrial oxidative stress, mitochondrial enzymes, and antioxidant defense system.

Parameters	Control	Mito-TEMPO	5-FU	5-FU + Mito-TEMPO
mtLPO (nmol/min/mg protein)	0.60 ± 0.01	0.59 ± 0.02	0.72 ± 0.02*⁣*^*∗*^	0.65 ± 0.02*⁣*^*∗*^^£#^
mtROS (relative intensity AFU)	157.36 ± 20.68	153.02 ± 9.68	203.46 ± 17.26*⁣*^*∗*^	165.84 ± 13.11^#^
MMP (relative intensity AFU)	534.80 ± 20.05	542.10 ± 25.60	380.10 ± 18.63*⁣*^*∗*^	468.86 ± 15.24*⁣*^*∗*^^£#^
Complex I (nmole NADH oxidized/min/mg mitochondrial protein)	1.87 ± 0.03	1.88 ± 0.02	0.99 ± 0.01*⁣*^*∗*^	1.56 ± 0.04*⁣*^*∗*^^£#^
Complex II (nmole/min/mg mitochondrial protein)	85.83 ± 3.57	85.43 ± 1.79	37.57 ± 2.96*⁣*^*∗*^	63.77 ± 5.25*⁣*^*∗*^^£#^
Complex IV (nmole NADH oxidized/min/mg mitochondrial protein)	0.43 ± 0.01	0.44 ± 0.01	0.16 ± 0.01*⁣*^*∗*^	0.30 ± 0.01*⁣*^*∗*^^£#^
IDH (nmole NADH oxidized/min/mg mitochondrial protein)	0.66 ± 0.01	0.67 ± 0.02	0.24 ± 0.02*⁣*^*∗*^	0.40 ± 0.01*⁣*^*∗*^^£#^
MDH (nmol NADH oxidized/min/mg mitochondrial protein)	88.99 ± 1.71	90.07 ± 4.19	40.69 ± 1.32*⁣*^*∗*^	66.75 ± 3.33*⁣*^*∗*^^£#^
mtGSH (nmol/mg mitochondrial protein)	0.61 ± 0.02	0.60 ± 0.02	0.33 ± 0.02*⁣*^*∗*^	0.46 ± 0.02*⁣*^*∗*^^£#^
mtGR (nmol/mg mitochondrial protein)	0.41 ± 0.01	0.42 ± 0.01	0.20 ± 0.01*⁣*^*∗*^	0.31 ± 0.01*⁣*^*∗*^^£#^
mtGPx (nmol/mg mitochondrial protein)	0.53 ± 0.05	0.63 ± 0.09	0.28 ± 0.03*⁣*^*∗*^	0.43 ± 0.06*⁣*^*∗*^^£#^
mtSOD (IU/mg mitochondrial protein)	0.17 ± 0.01	0.16 ± 0.02	0.07 ± 0.02*⁣*^*∗*^	0.13 ± 0.01*⁣*^*∗*^^£#^

*Note:* Data were expressed as mean ± SD and analyzed using one-way ANOVA followed by post hoc test (Tukey's HSD).

*⁣* ^*∗*^Represents *p* ≤ 0.05 when compared with the control group.

^£^Represents *p* ≤ 0.05 when compared with the Mito-TEMPO group.

^#^Represents *p* ≤ 0.05 when compared with the 5-FU group.

## Data Availability

Data are available and can be provided upon request to the corresponding author.
